# Paternal therapy with disease modifying drugs in multiple sclerosis and pregnancy outcomes: a prospective observational multicentric study

**DOI:** 10.1186/1471-2377-14-114

**Published:** 2014-05-26

**Authors:** Chiara Pecori, Marta Giannini, Emilio Portaccio, Angelo Ghezzi, Bahia Hakiki, Luisa Pastò, Lorenzo Razzolini, Andrea Sturchio, Laura De Giglio, Carlo Pozzilli, Damiano Paolicelli, Maria Trojano, Maria Giovanna Marrosu, Francesco Patti, Gian Luigi Mancardi, Claudio Solaro, Rocco Totaro, Maria Rosaria Tola, Giovanna De Luca, Alessandra Lugaresi, Lucia Moiola, Vittorio Martinelli, Giancarlo Comi, Maria Pia Amato

**Affiliations:** 1 Department of NEUROFARBA, Section Neurosciences, University of Florence, Largo Brambilla 3, 50134 Florence, Italy; 2 Multiple Sclerosis Center, S Antonio Abate Hospital, Gallarate, Italy; 3 Multiple Sclerosis Center, S Andrea Hospital, La Sapienza University, Rome, Italy; 4 Department of Neurology, University of Bari, Bari, Italy; 5 Multiple Sclerosis Center, Department of Neurology, University of Cagliari, Cagliari, Italy; 6 Multiple Sclerosis Center, University of Catania, Catania, Italy; 7 Department of Neurology, University of Genova, Genoa, Italy; 8 Department of Neurology, ASL3 Genovese, Genoa, Italy; 9 Department of Neurology, University of L’Aquila, L’Aquila, Italy; 10 Department of Neuroscience, University of Ferrara, Ferrara, Italy; 11 Department of Oncology and Neurosciences, Section of Neurology, University G. D'Annunzio Chieti, Chieti, Italy; 12 Department of Neurology, Scientific Institute H. San Raffaele, University of Milan, Milan, Italy; 13 Department of Clinical and Experimental Medicine, Neurology Unit, University of Pisa, Pisa, Italy

**Keywords:** Multiple sclerosis, Paternity, Pregnancy, Interferon beta, Glatiramer acetate

## Abstract

**Background:**

Most of Multiple Sclerosis (MS) patients undergo disease modifying drug (DMD) therapy at childbearing age. The objective of this prospective, collaborative study, was to assess outcomes of pregnancies fathered by MS patients undergoing DMD.

**Methods:**

Structured interviews on pregnancies fathered by MS patients gathered in the Italian Pregnancy Dataset were collected; pregnancies were divided according to father exposure or unexposure to DMD at time of procreation. Treatment were compared with multivariable logistic and linear models.

**Results:**

Seventy-eight pregnancies fathered by MS patients were tracked. Forty-five patients were taking DMD at time of conception (39 beta-interferons, 6 glatiramer acetate), while 33 pregnancies were unexposed to DMD. Seventy-five pregnancies ended in live-births, 44 in the exposed and 31 in the unexposed group. No significant differences between the two groups were found in the risk of spontaneous abortion or malformations (p > 0.454), mean gestational age (p = 0.513), frequency of cesarean delivery (p = 0.644), birth weight (p = 0.821) and birth length (p = 0.649). In comparison with data of the Italian general population, the proportion of spontaneous abortion and caesarean delivery in exposed pregnancies fell within the estimates, while the proportion of pre-term delivery in the exposed group was higher than expected.

**Conclusions:**

Our data indicate no association between paternal DMD exposure at time of conception and risk of spontaneous abortion, adverse fetal outcomes and congenital malformations. Further studies clarifying the role of DMD fathers intake prior and during pregnancy are desirable, to supply guidelines for clinical practice.

## Background

Multiple Sclerosis (MS) is the most frequent and important inflammatory demyelinating disease of the Central Nervous System (CNS) [1]. Since the disease mostly affects young people at childbearing age, great interest exists about the effects on pregnancy outcomes and health of the baby related with disease modifying drugs (DMD) taken around conception and during pregnancy. Over the past few years, a number of papers have addressed issues of safety of the DMDs at the time of procreation and during pregnancy in MS mothers [2]. In particular, prospective cohort studies observed no major adverse safety signals associated with Interferon-beta (IFN-beta) exposure, compared with outcomes of unexposed pregnancies in MS patients [3,4]. In a previous collaborative Italian study [3] exposure to IFN-beta during pregnancy was associated with lower mean birth weight, shorter mean birth length of the baby and shorter pregnancy duration. The differences, however, were minimal and without any major impact on children health. Despite limited sample size, studies on Glatiramer Acetate (GA) exposure also reported no strong safety signals, with the main fetal and pregnancy outcomes being comparable to those observed in the general population [5].

On the other hand, there is a dearth of information on the role of paternal DMD therapy on pregnancy outcome. Potential mechanisms of adverse effects may be due to direct mutagenic actions, impairment of spermatogenesis, or the transfer of chemicals via semen [6]. Expression of IFN receptors has been reported in the sperm cell [7] and cytokines and IFNs can have direct effects on spermatogenic cell differentiation and spermatogenesis [8]. Moreover, IFN-alfa has been found to impair spermatogenesis in a pre-clinical study [9]. As for the role of paternal DMD exposure in MS, a previous study [10] showed that MS-fathers taking DMD had new-borns with higher birth weight in comparison with children of MS-mothers (exposed or not to IFN-beta). Moreover, birth weight was only slightly lower in children of MS-fathers compared to a collective of healthy women [10]. On the basis of these findings, the authors concluded that MS-fathers’ DMD therapy is not associated with major risks on procreation. The study, however, was conducted in a small sample (32 males) and did not report a comparison with pregnancy outcomes in MS-fathers not exposed to DMD. A recently published study [11] addressed the association between DMD exposure and birth outcomes in 195 newborns of exposed and unexposed MS fathers. The authors found no compelling evidence to suggest that exposure was associated with either lower birth weight or gestational age [11].

The MS Study Group of the Italian Neurological Society launched a project on pregnancy in MS, establishing a network among the main national MS Centers. The dataset collected included information on pregnancy outcomes mothered and fathered by MS patients.

In this prospective, collaborative study, we focused on pregnancy and fetal outcomes related to MS patients who had fathered offspring while being treated with DMD.

## Methods

The Italian Pregnancy dataset has been described in detail elsewhere [3]. In brief, all pregnancies mothered and fathered by MS patients, diagnosed according to McDonald’s criteria [12] and referring to the participating Centers between 2002 and 2008 were identified and tracked over the whole gestational period. The 21 participating Italian MS Centers are located throughout the entire country; in each center patients were regularly followed-up every six months and in case of relapses. Demographic, clinical and therapeutic data were gathered by means of a standardized information form. Within six months after the end of pregnancy (delivery, miscarriage or abortion), patients were administered a semi-structured interview, dealing with pregnancy outcomes and potential confounders; children follow-up data were gathered after delivery and over a mean two-year period, considering that most malformations are usually identified during the first months of life.

The information form included a section related to MS patients (demographic data, education, smoke, alcohol intake, family history of malformations or other diseases, data regarding MS history and therapy), a section related to their partners’ pregnancy (maternal pharmacological therapies, *in utero* exposure to toxins or radiations, folic acid supplementation, maternal smoke, alcohol intake, abortion, pregnancy complications, amniocentesis, ecographic alterations), delivery and fetal outcomes (weeks of gestation, cesarean delivery, epidural anesthesia, neonatal complications, sex of the baby, birth weight and length, Apgar index at fifth minute, malformations). Conception was determined as 14 days after the mother’s last menstrual period. A specific section of the questionnaire investigated major developmental problems and malformations of the babies detected following birth, gathered and up-dated after delivery. In case of any malformation or major developmental problems were identified from the questionnaire, the baby’s clinical charts were reviewed.

Pregnancies were divided into two groups: those fathered by MS patients taking DMD at time of procreation or who had discontinued DMD within 70 days from conception (exposed pregnancies) and those fathered by MS patients who were not taking any medication, i.e. who had discontinued DMD at least 70 days from conception or who had never been treated (unexposed pregnancies).

Data on spontaneous abortion (before 22 completed weeks of gestation), pre-term delivery (before 37 weeks of gestation), cesarean delivery, baby birth weight and length were compared between DMD exposed and unexposed pregnancies; furthermore, the proportions of spontaneous abortion, pre-term and cesarean delivery were compared with data reported in the Italian population [13], derived from the Italian Statistical Institute (ISTAT) in 2005.

The study was approved by the Ethic Committee of the University of Florence and a written informed consent was obtained from all patients.

### Statistical analysis

Outcomes considered in statistical analysis were spontaneous abortion (yes vs no), pre-term delivery (yes vs no), cesarean delivery (yes vs no), birth weight and length of babies.

Baseline characteristics for treatment groups were reported as frequency (percentage) and mean ± standard deviation (SD), and compared with Pearson’s *χ*^2^, Fisher’s exact *χ*^2^ and Mann–Whitney U tests when appropriate.

Multivariable logistic and linear models were used for treatment comparisons, including as confounders factors related to MS fathers (including age at conception, education as an indicator of the socioeconomic status, disease duration), factors related to mothers (previous pregnancies and abortions [yes vs. no]), factors collected for both the father and the mother (smoking, alcohol and substance exposure during pregnancy [yes vs. no]), and factors related to delivery and fetal outcomes (gestational age, cesarean delivery, gender of the baby). In particular, a) the multivariable logistic model assessing spontaneous abortion as dependent variable included as confounders: age at conception, education, disease duration, previous pregnancies and abortions (yes vs. no), smoking, alcohol and substance exposure during pregnancy (yes vs. no). b) The multivariable logistic model assessing pre-term delivery as dependent variable included as confounders: age at conception, education, disease duration, previous pregnancies and abortions (yes vs. no), smoking, alcohol and substance exposure during pregnancy (yes vs. no), cesarean delivery, gender of the baby. c) The multivariable logistic model assessing caesarean delivery as dependent variable included as confounders: age at conception, education, disease duration, previous pregnancies and abortions (yes vs. no), smoking, alcohol and substance exposure during pregnancy (yes vs. no), gestational age, cesarean delivery, gender of the baby. d) The multivariable linear model assessing birth-weight as dependent variable included as confounders: age at conception, education, disease duration, previous pregnancies and abortions (yes vs. no), smoking, alcohol and substance exposure during pregnancy (yes vs. no), gestational age, cesarean delivery, gender of the baby. e) The multivariable linear model assessing birth-length as dependent variable included as confounders: age at conception, education, disease duration, previous pregnancies and abortions (yes vs. no), smoking, alcohol and substance exposure during pregnancy (yes vs. no), gestational age, cesarean delivery, gender of the baby.

Education missing data were substituted with the mean value of the study sample. Other missing data were deleted from the analysis.

Comparisons with Italian population data of proportions of spontaneous abortion, pre-term and cesarean delivery were performed using a one-sample binomial test with a 95% CI.

We used the SPSS 20.0 running on Windows (SPSS Chicago, IL, USA).

## Results

### Study sample

**Figure 1:**
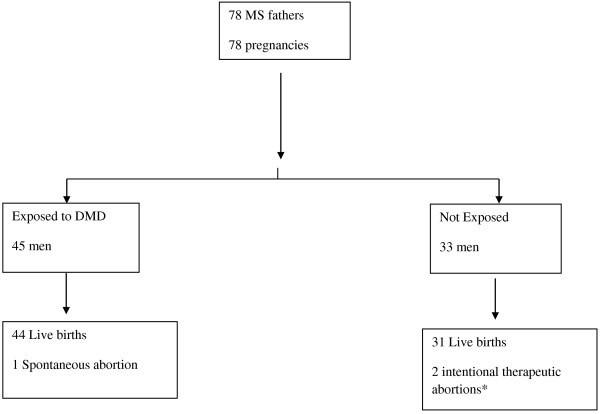
Flow-chart of the pregnancies enrolled in the study.

During the study period, a total of 78 pregnancies fathered by MS patients were tracked (Figure 1; Table 1). The median follow-up period after delivery was 1.8 years (range 0.1-10.7 years).

**Table 1:** Characteristics of the study sample

	**Total sample (#78)**	**Exposed to DMD (#45)**	**Not-exposed (#33)**	**p**
*Variables related to MS father*				
Age, mean (SD) years (#78)	34.8 (4.8)	35.4 (5.3)	33.9 (3.9)	0.476*
Education, mean (SD) years (#62)	13.4 (3.5)	13.2 (3.3)	13.8 (4.0)	0.548*
Age at onset, mean (SD) years (#75)	26.5(6.8)	27.0 (7.2)	25.7 (6.2)	0.352*
Disease duration, mean (SD) years (#74)	8.4 (5.9)	8.5 (6.0)	8.3 (5.9)	0.812*
Smokers # (%) (#78)	26 (33.3)	14 (31.1)	12 (36.4)	0.627**
Alcohol # (%) (#78)	14 (17.9)	10 (22.2)	4 (12.1)	0.251**
*Variables related to pregnancy and fetal outcomes*				
Drug and toxin exposure # (%) (78)	-	-	-	-
Smoking exposure # (%) (78)	5 (6.4)	2 (4.4)	3 (9.1)	0.645§
Alcohol exposure # (%) (#78)	-	-	-	-
Gestational age (weeks) (#72)	38.9 (3.8)	39.0 (2.1)	38.7 (5.2)	0.513*
Preterm delivery # (%) (#72)	10 (13.9)	7 (17.1)	3 (9.7)	0.499§
Caesarean delivery # (%) (#75)	24 (32.0)	15 (34.1)	9 (29.0)	0.644**
Epidural analgesia # (%) (#75)	16 (21.3)	10 (22.7)	6 (19.4)	0.726**
Gender of the baby male/female (#75)	36/39	21/23	15/16	0.955**
Birth-weight mean (SD) gr (#75)	3301.3 (454.9)	3282.3 (445.5)	3328.4 (474.1)	0.821*
Birth-length mean (SD) cm (#67)	50.1 (2.2)	49.9 (2.3)	50.2 (2.1)	0.649*

DMD: Disease Modifying Drug; SD: Standard Deviation.

*Mann Whitney *U*-test; ** *χ*^2^ test; §Fisher’s exact *χ*^2^ test.

p values related to the exposed versus unexposed group comparison.

Forty-five patients (57.7%) were taking DMD around the time of conception (17 subcutaneous interferon Beta-1a (IFNB-1a), 14 intramuscular IFNB-1a, 8 subcutaneous IFNB-1b, 6 glatiramer acetate (GA) (Table 1).

The group of 33 unexposed pregnancies included those fathered by MS patients who had never been treated with DMD (27 subjects) or had interrupted DMD intake at least 70 days before conception (within six months in six subjects).

Twenty-six patients were classified as smokers, 14 in the exposed and 12 in the unexposed group. Furthermore, 14 patients were customary alcohol consumers, 10 in the exposed and 4 in the unexposed group.

In pregnant partners of patients’ group there were five smokers, two in the exposed and three in the unexposed pregnancy group, while no women reported either alcohol intake or drug and toxin exposure during pregnancy. Five women had had previous pregnancies (two exposed, three unexposed) and two had had previous abortions (one exposed, one unexposed).

### Abortion and fetal malformations

A total of 78 pregnancies were analyzed: 45 exposed and 33 unexposed. We observed 75 pregnancies resulting in live-births (36 males, 39 females), 44 in the exposed and 31 in the unexposed pregnancy group. One pregnancy of the exposed group ended in spontaneous abortion, occurring after approximately 12 weeks of gestation (Figure 1).

Three (6.7%) fetal malformations or complications were observed in the exposed group (scalp hemangioma, esophageal atresia, jaundice and hip dysplasia), while four (12.1%) occurred in the unexposed group: Edward Syndrome, Down Syndrome, both leading to intentional therapeutic abortion, one case of ocular malformation (myopia and cataract) and one of renal agenesis (solitary kidney) (Table 2).

**Table 2:** Fetal malformations/complications

	**Exposed pregnancies**	**Unexposed pregnancies**
Fetal malformation/complications (p = 0.454)*	1 Scalp hemangioma	1 Edward Syndrome
	1 Esophageal atresia	1 Down Syndrome
	1 Jaundice and hip dysplasia	1 Ocular malformation (myopia and cataract)
		1 Renal agenesis (solitary kidney)

*Fisher’s exact *χ*^2^ test.

There was no significant association between DMD father exposure and risk of spontaneous abortion or malformations (Fisher’s exact *χ*^2^ test p > 0.454).

### Pre-term and cesarean delivery

Mean gestational age was 39.0 ± 2.1 weeks in exposed pregnancies and 38.7 ± 5.2 in the unexposed ones (p = 0.513). Pre-term delivery was observed in seven exposed pregnancies (17.1% of live birth deliveries) and three unexposed pregnancies (9.7% of live birth deliveries; p = 0.499). Pre-term delivery was observed at 32 and at 33 weeks in two cases, at 36 weeks for two babies and at 37 weeks for three babies in exposed pregnancies. The three preterm unexposed pregnancies ended at 35, 36 and 37 weeks respectively.

In multivariable analysis, cesarean delivery (OR = 4.41, 95% CI 1.00-20.50; p = 0.050) was the only predictor of pre-term delivery.

Cesarean delivery was performed in 15 exposed (34.1% of live birth deliveries) and 9 unexposed pregnancies (29.0% of live birth delivery; p = 0.644) and was associated with lower gestational age (OR = 0.74; 95% CI 0.55-0.99; p = 0.040) in multivariable analysis.

### Birth weight and length

Mean birth-weight of newborns was 3.282 ± 445 g in exposed and 3.328 ± 474 g in unexposed pregnancies (p = 0.821).

Mean birth-length was 49.9 ± 2.3 cm in the exposed and 50.2 ± 2.1 cm in the unexposed group (p = 0.649).

In multivariable analysis, father’s higher education (school years) and lower age at conception were associated with lower birth-weight (Table 3). Higher education predicted shorter birth-length (Table 3).

**Table 3:** Significant predictors of birth-weight and birth-length in live birth pregnancies

	**B**	**p**
**Birth-weight**		
Education*	−35.1	0.048
Age at conception*	22.4	0.038
**Birth-length**		
Education*	−0.25	0.004

Multivariable linear regression models including as confounders: age at conception, education, disease duration, previous pregnancies and abortions (yes vs. no), smoking, alcohol and substance exposure during pregnancy (yes vs. no), gestational age, cesarean delivery, gender of the baby.

*related to the father.

### Pregnancy complications and follow up of the babies

Maternal complications occurred in seven out of 44 live birth pregnancies in the exposed group (15.9%) and in one out of 31 live birth pregnancies (3.2%) in the unexposed group (p = 0.130; Table 4). In the exposed group, we observed two cases of preeclampsia, one partial placental abruption, two threatened abortion, one threatened preterm delivery and a minor infection, while in the unexposed group we found one case of placental abruption.

**Table 4:** Maternal complications in pregnancies where the father was exposed or unexposed to a DMD for MS at the time of conception

	**Exposed pregnancies**	**Unexposed pregnancies**
Maternal complications (p = 0.130)	2 Pre-eclampsia/eclampsia	1 Placental abruption
	2 Threatened abortion	
	1 Threatened preterm delivery	
	1 Partial placental abruption	
	1 Minor infection	

*Fisher’s exact *χ*^2^ test.

In a median follow-up period of 1.8 years (range 0.1-10.7), we observed one developmental abnormality (mild speech disorder) in a child of the unexposed group.

### Comparison between exposed pregnancies and Italian general population data

During the year 2005, in the Italian population, a total of 128.09 spontaneous abortions every 1,000 live-births were observed [13]. Hypothesizing the same incidence in our cohort, the 95% CI expected proportion of spontaneous abortions in the exposed group was 1.5%–20.5%. In our sample the observed proportion of spontaneous abortion was one in 44 live-births, which fell within the estimates.

Pre-term delivery was observed in 6.7% of live births and caesarean delivery in 37.3% [13]. Hypothesizing the same incidence, the 95% CI expected proportion of pre-term delivery in the exposed group was 0%–14.3%, and the 95% CI expected proportion of caesarean delivery was 22.7%-51.9%. The proportion of pre-term delivery observed in the exposed group (17.1%) was higher than expected, whereas the proportion of caesarean delivery (34.1%) fell within the estimates.

## Discussion

Increasing interest has been posed on MS patients parenthood, and in particular on DMD exposure around conception and early pregnancy [14]. Previous studies have not reported any major adverse safety signals associated with the mothers exposure to interferon beta or glatiramer acetate on risk of obstetric/neonatal complications and congenital malformations [3-5], although some studies have suggested potential harm of interferon beta in terms of lower mean birth weight and length and preterm birth [2].

The information on pregnancy and fetal outcomes of MS-fathers is limited. In a recent study, paternal MS and MS-related clinical factors were not significantly associated with birth outcomes [15]. As for MS-fathers’ exposure to DMD, previous studies suggest that in paternity by MS patients under DMD no major adverse safety signals were found [10,11]. No differences in the risk of spontaneous abortion and congenital anomalies, birth weights and birth lengths was observed between father-exposed pregnancies and data obtained from the general population; paternity of MS-fathers under DMD resulted in only slightly lower birth weight compared to a collective of healthy women [10]. This finding was not confirmed in a recent study on a larger sample, in which IFNβ or GA exposure around the time of conception was not associated with either lower birth weight or gestational age [11].

The interest on paternally-mediated adverse pregnancy outcomes comes from the notion that drug intake around conception could cause reproductive toxicity. Three main mechanisms have been described: non-genetic (due to the presence of a drug in seminal fluid), genetic (gene mutation or chromosomal abnormality), and epigenetic (effect on gene expression, genomic imprinting, or DNA methylation) [16,17]. The development of spermatozoa from germ cells takes approximately 64 days, and a further 2–5 days are required for spermatozoa to pass through the epididymis [16,18]. This period of intense cellular transformation could be considered to be highly susceptible to environmental insults, including drug exposure. Therefore, in our study, we considered as exposed those pregnancies fathered by MS patients who were taking DMD at conception or had discontinued DMD within 70 days before conception.

Interferon beta and glatiramer acetate have shown no mutagenic effects on *in vitro* studies [19,20]. To date, information on interferon beta and glatiramer acetate seminal fluid concentration and epigenetic effect on offspring after paternal exposure are lacking.

In the present study we examined 78 pregnancies fathered by MS patients, 45 exposed to DMD therapy at time of conception.

We found no significant differences in the risk of spontaneous abortion and malformations, mean gestational age, frequency of cesarean delivery, birth weight and birth length between DMD exposed and unexposed pregnancies. Moreover, comparing data of exposed pregnancies with incidence estimates inferred from the Italian general population [13], we found no significant association between DMD exposure and risk of spontaneous abortion and cesarean delivery.

Although we found no significant difference in preterm delivery rates between exposed and unexposed pregnancies, the proportion of pre-term delivery observed in the exposed group (17.1%) was higher than expected, considering the estimates extrapolated from general population.

There were no significant differences in the risk of maternal complications, among which partial placental abruption, preeclampsia, threatened abortion, threatened preterm delivery, and a minor infection. However, the considerable heterogeneity of the observed maternal complications in our small study sample, does not allow to draw definitive conclusions.

Eventually, in our study sample, data on possible developmental abnormalities are encouraging, because in a median follow-up period of 1.8 years we only observed the occurrence of mild speech disorders in a child in the unexposed pregnancies group.

Some limitations have to be considered in the interpretation of the study’s results. The study sample was relatively small, and some data of patients' partner were lacking (for instance maternal age at conception). Moreover, since the questionnaire collected very detailed complex medical data directly for the fathers a recall bias cannot be excluded. Furthermore, we had no information on sexual habits, and we cannot infer the influence of paternal exposure during pregnancy related to potential *in utero* fetal exposure. Finally, as for the comparison of the Italian population, demographic matching cannot be warranted, since information on age of the mothers was lacking.

## Conclusions

Our data suggest the absence of association between paternal DMD exposure at time of conception and risk of spontaneous abortion, adverse fetal outcomes and congenital malformations. Although we found a higher preterm delivery rate in the exposed group, these data should be interpreted with caution in light of the above considerations. Further studies addressing the issue of MS patients paternity are desirable, to better clarify the role of DMD fathers intake prior and during pregnancy and to supply guidelines for clinical practice purposes.

## Abbreviations

MS: Multiple sclerosis; CNS: Central nervous system; DMD: Disease modifying drug; IFN-beta: Interferon beta; GA: Glatiramer acetate.

## Competing interests

Dr. Pecori receives research support from Novartis and FISM (Fondazione Italiana Sclerosi Multipla). Dr. Giannini has nothing to disclose. Dr. Portaccio serves on a scientific advisory board for Biogen-Idec, Merck Serono and Bayer, received honoraria for speaking from Biogen-Idec and Teva, and receives research support from Merck Serono, Biogen-Idec, Bayer Schering Pharma and Sanofi Aventis. Dr. Ghezzi serves on scientific advisory boards for Merck Serono and Teva Pharmaceutical Industries ltd; received honoraria for speaking form Merck Serono, Biogen Idec, Bayer Schering Pharma, and Novartis; serves as a consultant for Novartis, receives research support from Sanofi Aventis, Biogen Idec and Merck Serono. Dr. Hakiki received research support. from Merck Serono, Novartis and Teva. Dr. Pastò has nothing to disclose. Dr. Razzolini has nothing to disclose. Dr. Sturchio has nothing to disclose. Dr. De Giglio has nothing to disclose. Dr. Pozzilli serves on advisory boards for and received speaker honoraria from Sanofi Aventis, Biogen, Bayer Schering Pharma, Novartis, Teva, Merck Serono and research grants from Merck Serono, Sanofi Aventis, Novartis and Biogen. Dr. Paolicelli serves as a consultant for Merk Serono and Bayer Schering Pharma. Dr. Trojano received honoraria for consultancy or speaking from Sanofi-Aventis, Biogen, and Bayer Schering, and research support from Merck Serono and Biogen Idec. Dr. Marrosu serves on a scientific advisory board for Merck Serono, Biogen Idec and Bayer Schering Pharma; has received funding for travel from Biogen Idec, Merk Serono, Bayer Shering Pharma and Sanofi Aventis; serves on the editorial board of Neurological Sciences; has received speakers honoraria from Biogen Idec and Merk Serono; has received research support from Biogen-Idec, Merck Serono, Fondazione Banco di Sardegna. Dr. Patti served on a scientific advisory board for Bayer Shering Pharma, Biogen-Idec, Merck Serono, Novartis, and Sanofi Aventis, received honoraria for speaking from Bayer, Biogen-Idec, Merck Serono, Novartis, and Sanofi Aventis and funding for travel from Merck Serono and TEVA, has received research support from the University of Catania and FISM. Dr. Mancardi has received funding for travel from Biogen Idec, Merk Serono and Bayer Shering Pharma; speaker honoraria from Bayer Schering Pharma, Biogen Idec, Sanofi-Aventis, and Novartis; serves on the editorial board of Neurological Sciences. Dr. Solaro has nothing to disclose. Dr. Totaro has received honoraria for consultancy or speaking from Bayer-Schering Pharma, Biogen Idec, Merck Serono, Novartis Pharma, Sanofi-Aventis, and Teva. DR. Tola has served on scientific advisory boards and received speakers honoraria from Biogen Idec, Sanofi Aventis, Merk Serono and Novartis and has received research support from Sanofi Aventis. Dr. De Luca has nothing to disclose. Dr Lugaresi has served on scientific advisory boards for Bayer Schering, Biogen Idec, Genzyme, and Merck Serono, received travel grants and honoraria from Bayer Schering, Biogen Idec, Merck Serono, Novartis, Sanofi Aventis and Teva Pharmaceutical Industries; has received research support from Bayer Schering Pharma, Biogen Idec, Merck Serono, Novartis, Sanofi Aventis, Teva, Associazione Italiana Sclerosi Multipla (AISM), serves as a Consultant for “Fondazione Cesare Serono”. Dr. Moiola has nothing to disclose. Dr. Martinelli has received speaker honoraria and funding for travel from Biogen-Idec, Merck Serono, Bayer Schering Pharma, Novartis and Sanofi Aventis and has served as a consultant to Bayer Schering Pharma, Sanofi Aventis and Teva Pharmaceutical Industries. Dr. Comi serves on scientific advisory boards for Bayer Schering Pharma, Merck Serono, Teva Pharmaceutical Industries Ltd, Sanofi Aventis, Novartis and Biogen-Idec; has received speaker honoraria from Teva Pharmaceutical Industries Ltd, Sanofi Aventis, Serono Sumposia International Foundation, Biogen-Idec, Merck Serono, Novartis, Bayer Schering and Sanofi Aventis. Dr. Amato serves on scientific advisory boards for, has received speakers honoraria and research support from Biogen-Idec, Merck Serono, Bayer Schering Pharma and Sanofi Aventis and serves on the editorial board of BMC Neurology.

## Authors’ contributions

CP: Drafting/revising the manuscript. Study concept or design. Analysis or interpretation of data. Acquisition of data. MG: Drafting/revising the manuscript. Study concept or design. Acquisition of data. EP: Drafting/revising the manuscript. Study concept or design. Analysis or interpretation of data. Acquisition of data. Statistical analysis. Study supervision. AG: Study concept or design. Acquisition of data. Study supervision. BH: Drafting/revising the manuscript. Study concept or design. Acquisition of data. LP: Drafting/revising the manuscript. Study concept or design. Acquisition of data. LR: Study concept or design. Acquisition of data. AS: Study concept or design. Acquisition of data. LDG: Study concept or design. Acquisition of data. CP: Study concept or design. Acquisition of data. Study supervision. DP: Study concept or design. Acquisition of data. MT: Study concept or design. Acquisition of data. Study supervision. MGM: Study concept or design. Acquisition of data. Study supervision. FP: Study concept or design. Acquisition of data. Study supervision. GLM: Study concept or design. Acquisition of data. Study supervision. CS: Study concept or design. Acquisition of data. Study supervision. RT: Study concept or design. Acquisition of data. Study supervision. MRT: Study concept or design. Acquisition of data. Study supervision. GDL: Study concept or design. Acquisition of data. AL: Study concept or design. Acquisition of data. LM: Study concept or design. Acquisition of data. VM: Study concept or design. Acquisition of data. GC: Study concept or design. Acquisition of data. Study supervision. MPA: Drafting/revising the manuscript. Study concept or design. Analysis or interpretation of data. Study supervision. All authors read and approved the final manuscript.

## Pre-publication history

The pre-publication history for this paper can be accessed here:

http://www.biomedcentral.com/1471-2377/14/114/prepub

## References

[B1] Noseworthy JH Lucchinetti C Rodriguez M Weinshenker BG Multiple sclerosis N Engl J Med 2000 343 938 952 10.1056/NEJM200009283431307 11006371

[B2] Lu E Wang BW Guimond C Synnes A Sadovnick D Tremlett H Disease-modifying drugs for multiple sclerosis in pregnancy: a systematic review Neurology 2012 11 1130 1135 22933738 10.1212/WNL.0b013e3182698c64 PMC3525300

[B3] Amato MP Portaccio E Ghezzi A Hakiki B Zipoli V Martinelli V Moiola L Patti F La Mantia L Mancardi GL Solaro C Tola MR Pozzilli C De Giglio L Totaro R Lugaresi A Di Tommaso V Paolicelli D Marrosu MG Comi G Pellegrini F Trojano M MS Study Group of the Italian Neurological Society Pregnancy and fetal outcomes after interferon-β exposure in multiple sclerosis Neurology 2010 20 1794 1802 21079181 10.1212/WNL.0b013e3181fd62bb

[B4] Sandberg-Wollheim M Alteri E Moraga MS Kornmann G Pregnancy outcomes in multiple sclerosis following subcutaneous interferon beta-1a therapy Mult Scler 2011 4 423 430 21220368 10.1177/1352458510394610

[B5] Giannini M Portaccio E Ghezzi A Hakiki B Pastò L Razzolini L Piscolla E De Giglio L Pozzilli C Paolicelli D Trojano M Marrosu MG Patti F La Mantia L Mancardi G Solaro C Totaro R Tola MR De Luca G Lugaresi A Moiola L Martinelli V Comi G Amato MP Pregnancy and fetal outcomes after Glatiramer Acetate exposure in patients with multiple sclerosis: a prospective observational multicentric study BMC Neurol 2012 12 124 10.1186/1471-2377-12-124 23088447 PMC3487812

[B6] Lee CYW Jin C Mata AM Tanaka T Einarson A Koren G A pilot study of paternal drug exposure: the motherisk experience Reprod Toxicol 2010 29 353 360 10.1016/j.reprotox.2010.01.008 20096774

[B7] Naz RK Chauhan SC Rose LP Expression of alpha and gamma interferon receptors in the sperm cell Mol Reprod Dev 2000 56 2 189 197 10.1002/(SICI)1098-2795(200006)56:2<189::AID-MRD10>3.0.CO;2-M 10813851

[B8] Hedger MP Meinhardt A Cytokines and the immune-testicular axis J Reprod Immunol 2003 58 1 1 26 10.1016/S0165-0378(02)00060-8 12609522

[B9] Ulusoy E Cayan S Yilmaz N Aktaş S Acar D Doruk E Interferon alpha-2b may impair testicular histology including spermatogenesis in a rat model Arch Androl 2004 50 5 379 385 10.1080/01485010490474823 15551753

[B10] Hellwig K Haghikia A Gold R Parenthood and immunomodulation in patients with multiple sclerosis J Neurol 2010 257 580 583 10.1007/s00415-009-5376-z 19936821

[B11] Lu E Zhu F Zhao Y van der Kop M Synnes A Dahlgren L Sadovnick AD Traboulsee A Tremlett H Birth outcomes in newborns fathered by men with multiple sclerosis exposed to disease-modifying drugs CNS Drugs 2014 28 5 475 482 10.1007/s40263-014-0154-6 24643915

[B12] McDonald WI Compston A Edan G Goodkin D Hartung HP Lublin FD McFarland HF Paty DW Polman CH Reingold SC Sandberg-Wollheim M Sibley W Thompson A van den Noort S Weinshenker BY Wolinsky JS Recommended diagnostic criteria for multiple sclerosis: guidelines from the international panel on the diagnosis of multiple sclerosis Ann Neurol 2001 50 121 127 10.1002/ana.1032 11456302

[B13] ISTAT Dimissioni dagli istituti di cura per aborto spontaneo in Italia [http://www.istat.it/dati/dataset/20080415_01/]

[B14] Finkelsztejn A Brooks JB Paschoal FM Jr Fragoso YD What can we really tell women with multiple sclerosis regarding pregnancy? A systematic review and meta-analysis of the literature BJOG 2011 118 790 797 10.1111/j.1471-0528.2011.02931.x 21401856

[B15] Lu E Zhu F Zhao Y van der Kop M Sadovnick A Synnes A Dahlgren L Traboulsee A Tremlett H Birth outcomes of pregnancies fathered by men with multiple sclerosis Mult Scler 2014 in press 10.1177/1352458514521308 24500603

[B16] Trasler JM Doerksen T Teratogen update: paternal exposures-reproductive risks Teratology 1999 60 161 172 10.1002/(SICI)1096-9926(199909)60:3<161::AID-TERA12>3.0.CO;2-A 10471901

[B17] De Santis M Cesari E Cavaliere A Ligato MS Nobili E Visconti D Caruso A Paternal exposure and counselling: experience of a teratology information service Reprod Toxicol 2008 26 42 46 10.1016/j.reprotox.2008.06.003 18598753

[B18] Cordier S Evidence for a role of paternal exposures in developmental toxicity Basic Clin Pharmacol Toxicol 2008 102 176 181 10.1111/j.1742-7843.2007.00162.x 18226072

[B19] Shimada H Ebine Y Kurosawa Y Arauchi T Mutagenicity studies of human fibroblast interferon (HuIFN-beta) Mutat Res 1984 139 183 187 10.1016/0165-7992(84)90125-8 6371521

[B20] TEVA Copaxone® full prescribing information [http://copaxone.com/pdfs/PrescribingInformation.aspx]

